# Study of Linkage between Glutathione Pathway and the Antibiotic Resistance of *Escherichia coli* from Patients’ Swabs

**DOI:** 10.3390/ijms16047210

**Published:** 2015-03-31

**Authors:** Marketa Kominkova, Petr Michalek, Kristyna Cihalova, Roman Guran, Natalia Cernei, Lukas Nejdl, Kristyna Smerkova, Simona Dostalova, Dagmar Chudobova, Zbynek Heger, Radek Vesely, Jaromir Gumulec, Jindrich Kynicky, Kledi Xhaxhiu, Ondrej Zitka, Vojtech Adam, Rene Kizek

**Affiliations:** 1 Department of Chemistry and Biochemistry, Faculty of Agronomy, Mendel University in Brno, Zemedelska 1, CZ-613 00 Brno, Czech Republic; E-Mails: kominkova.marketa@gmail.com (M.K.); petrmichalek85@gmail.com (P.M.); kriki.cihalova@seznam.cz (K.C.); r.guran@email.cz (R.G.); cernei.natalia3@gmail.com (N.C.); lukasnejdl@gmail.com (L.N.); k.smerkova@gmail.com (K.S.); esedinka@seznam.cz (S.D.); dagmar.chudobova@centrum.cz (D.C.); heger@mendelu.cz (Z.H.); zitkao@seznam.cz (O.Z.); vojtech.adam@mendelu.cz (V.A.); 2 Central European Institute of Technology, Brno University of Technology, Technicka 3058/10, CZ-616 00 Brno, Czech Republic; E-Mails: j.gumulec@gmail.com (J.G.); kledi.xhaxhiu@unitir.edu.al (K.X.); 3 Clinic of Traumatology at the Medical Faculty, Masaryk University of Brno, Ponavka 6, CZ-662 50 Brno, Czech Republic; E-Mail: r.vesely@unbr.cz; 4 Department of Pathological Physiology, Faculty of Medicine, Masaryk University, Komenskeho Namesti 2, CZ-662 43 Brno, Czech Republic; 5 Karel Englis College, Sujanovo Nam. 356/1, CZ-602 00 Brno, Czech Republic; E-Mail: jindrak@email.cz

**Keywords:** buthionine sulfoximine, *Escherichia coli*, glutathione, infections, swabs

## Abstract

In this work, we focused on the differences between bacterial cultures of *E. coli* obtained from swabs of infectious wounds of patients compared to laboratory *E. coli.* In addition, blocking of the protein responsible for the synthesis of glutathione (γ-glutamylcysteine synthase—GCL) using 10 mM buthionine sulfoximine was investigated. Each *E. coli* showed significant differences in resistance to antibiotics. According to the determined resistance, *E. coli* were divided into experimental groups based on a statistical evaluation of their properties as more resistant and more sensitive. These groups were also used for finding the differences in a dependence of the glutathione pathway on resistance to antibiotics. More sensitive *E. coli* showed the same kinetics of glutathione synthesis while blocking GCL (*K*_m_ 0.1 µM), as compared to non-blocking. In addition, the most frequent mutations in genes of glutathione synthetase, glutathione peroxidase and glutathione reductase were observed in this group compared to laboratory *E.coli*. The group of “more resistant” *E. coli* exhibited differences in *K*_m_ between 0.3 and 0.8 µM. The number of mutations compared to the laboratory *E. coli* was substantially lower compared to the other group.

## 1. Introduction

Microorganisms are normally present on epithelial linings as a physiological microflora, which acts as a barrier against colonization of potentially pathogenic microorganisms [1]. Once the outer body surface is invasively disintegrated, the microbes can enter the body tissues with deleterious effects. To avoid entering of the bacteria deeper into the organism, the healthy individuals trigger a machinery of defense mechanisms including local-dependent creation of blood proteins and phagocytes [2]. The fibrin coagulation helps to create a barrier against the microbe and prevents their penetration to the healthy tissue. The temperature rises due to the numerous defense processes, stimulated for the desired inflammation based on pathogen recognition by inner immune system [3].

Infection on the body surface, during the open wound, is a result of interaction between patient as the host, potential pathogen and the environment. Simply said, all these three factors affect the prognosis of the healing of the wound.

The identification of bacteria species in the wound, in the early stage of the infection, is crucial for subsequent treatment efficiency. Some novel methods and approaches have been recently published [4,5,6]. But if we look at the way the body copes with invasive bacterial infection we find that the phagocytes employ oxygen species as a weapon to kill the bacteria [2,7]. In this mechanism called a “respiratory burst”, the substances as superoxide, hydrogen peroxide or singlet oxygen are produced and transported to the phagosome to cause the deleterious imbalance of homeostasis in phagocytosed bacteria [8]. The temporary oxidative environment in the phagosome is strong enough to overcome the antioxidant defense of the bacteria. Thus, a question may arise, why the antioxidative mechanisms in such widespread bacteria as *E. coli* are so weak against this evolutionary pressure? To answer this question we need to focus on the background of general antioxidative defense mechanisms. In living organisms, one of the main antioxidants is glutathione (GSH).

**Figure 1:**
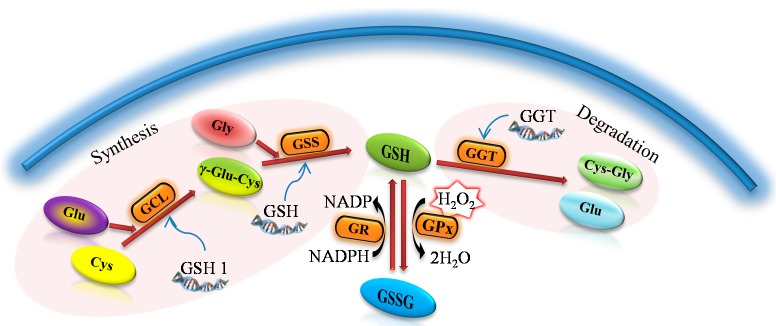
A general procedure for the synthesis and metabolism of glutathione. γ-glutamylcysteine (γ-Glu-Cys) arises due to the γ-glutamylcysteine synthase (GCL) from glutamic acid (Glu) and cysteine (Cys). The reduced form of glutathion (GSH) is synthesized from GCL and glycine (Gly) due to the glutathione synthase (GSS). As an antioxidant, GSH is oxidized to form oxidized glutathione (GSSG) with the participation of the glutathione peroxidase (GPx). Thanks to the action of glutathione reductase (GR) glutathione occurs primarily in the form of GSH. γ-glutamyl transpeptidase (GGT) causes the glutathione degradation in the cells to glutamic acid (Glu) and dipeptide cysteinylglycine (Cys-Gly).

The first mention of the tripeptide GSH (γ-l-glutamyl-l-cysteinyl-glycine) dates to 1888, when its presence was demonstrated in yeast [9,10]. Subsequently it was found that GSH has a number of important physiological functions and belongs to among the most abundant thiol compounds [11,12]. It exhibits antioxidant properties, protects against oxidative stress, maintains the redox balance of the cells, and acts as a cofactor for the enzymatic antioxidants [13]. An important property is also the detoxification of xenobiotics [14,15]. It can be found in all eukaryotic systems as well as in a large group of Gram-negative bacteria. However, the occurrence of GSH in Gram-positive cells is a complicated issue [16,17]. In plant and animal cells, GSH is generally synthesized (Figure 1) due to the two ATP-dependent enzymes in a metabolic pathway similar to all organisms throughout evolution [18]. The enzyme γ-glutamylcysteine synthase (GCL), which catalyzes the synthesis of the first intermediate in the synthesis of glutathione—glutamylcysteine (γ-Glu-Cys)—is considered, together with the availability of cysteine, as a limiting factor for the entire synthesis [19,20].

Similarly to eukaryotes, many prokaryotic cells, particularly Gram-negative bacteria, synthesize GSH. Production of this low molecular weight thiol compound is lower compared to the eukaryotes. Furthermore, GSH in some prokaryotic systems can be imported from the extracellular space and used for cellular reactions [21].

Commonly encountered infected wounds raise issues of diagnosis and treatment in medical practice because of the selection of bacterial strains resistant to antibiotics [22]. Chronic wounds are polymicrobial in nature, and thus pathogens will vary depending on the wound type with species from *Staphylococcus*, *Enterococcus*, *Enterobacter*, *Pseudomonas*, *Finegoldia genera* or *Escherichia coli* being the most commonly isolated from all types of wounds [22,23].

In this article, the differences in the antibiotic resistance between *E. coli* strains obtained from patients’ wound swabs and commercially available *E. coli* strains were evaluated. Also, changes in the protein profiles, enzymatic activity and gene expression and sequences after addition of buthionine sulfoximine (BSO), which is a specific blocker of synthesis of GCL, were tested. After blocking GCL, the synthesis of GSH is stopped. Our aim was to detect the differences in the GSH pathway of *E. coli*. Particular attention was paid to the resistance to a wide range of antibiotics.

## 2. Results and Discussion

*E. coli* belongs to the *Enterobacteriaceae* family, which commonly occurs as commensals of the digestive tract of humans and warm-blooded animals. It is also one of the most common causes of a broad spectrum of naturally occurring infections [24]. To observe the changes in properties after blocking the protein responsible for the GSH synthesis (GCL), the bacterial cultures of *E. coli* obtained from swabs of infected wounds from patients and from laboratory *E. coli* (always marked as number 1) were used. Further, we tried to describe one of the mechanisms that could affect the resistance of *E. coli* to commonly used antibiotics.

### 2.1. Effect of Buthionine Sulfoximine (BSO)

In the first part of the experiment, we attempted to characterize the behavior of bacterial cultures in relation to BSO. The synthesis of GSH is blocked, because this enzyme catalyzes the synthesis of γ-glutamylcysteine from which the GSH is synthesized by connection of glycine [25]. The effect of BSO on different strains of *E. coli* has been characterized by growth curves and by the calculation of minimum inhibitory concentrations after 24-h incubation (24IC_50_). The results of analysis are shown in Table 1.

**Table 1:** IC_50_ of buthionine sulfoximine (BSO) determined for *E. coli*. IC_50_ was determined from 24-h growth curves of *E. coli* with the applied BSO.

Sample No.	24IC_50_ (mM)
1	25
2794	21
2252	16
2873	21
2642	25
2552	15
2250	17
2654	15
2869	24

During the evaluation of differences in biochemical pathways of synthesis, oxidation, and reduction of GSH, we analyzed the biochemical properties of the *E. coli* strains and changes of these properties after blocking the pathway of GSH synthesis as well. We always compared *E. coli* strains after cultivation in the pure medium and *E. coli* strains after 6-h cultivation in the medium with addition of 10 mM BSO.

### 2.2. Changes in Protein Mass Profiles

**Figure 2:**
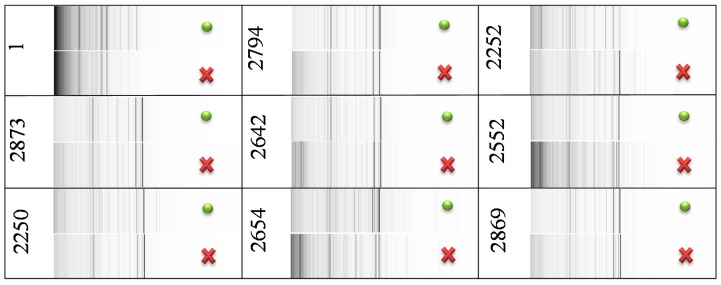
Comparison of MS profiles in pseudo-gel view of control *E. coli* (green dot) and of *E. coli* with blocked synthesis of GSH (red cross). The peak intensity is expressed in a gray scale—darker color of peak indicates higher peak intensity. The utilized mass range was 1–20 kDa.

Matrix-assisted laser desorption/ionization time-of-flight mass spectrometry (MALDI-TOF MS) of control *E. coli* samples and of *E. coli* samples with blocked GSH synthesis were compared using pseudo-gel views (Figure 2). This comparison showed that mass spectra of the samples differ only in peak intensities—no differential peaks were observed. Therefore, the addition of BSO caused no qualitative change in *E. coli* protein mass profiles, but it caused changes in peak intensities. It points at a large influence of BSO on proteosynthesis of tested strains, likely because of GSH pathway disruption.

### 2.3. Change in Antibiotic Resistance

**Figure 3:**
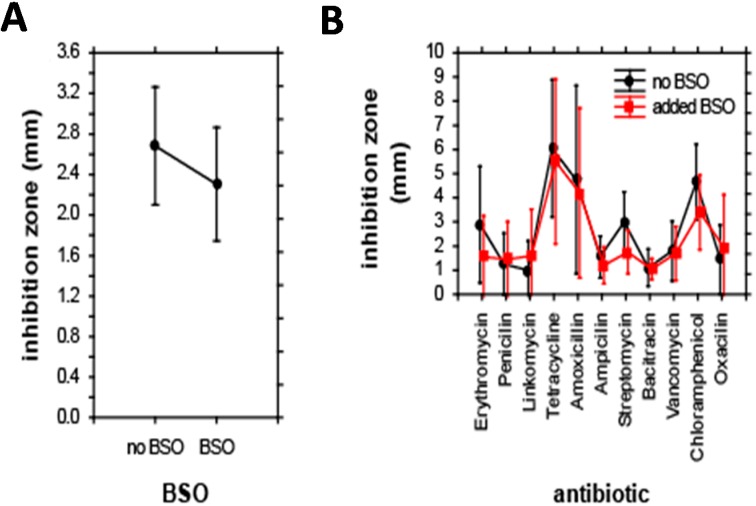
Effect of individual antibiotics on the size of inhibition zones. (**A**) Effect of BSO addition; and (**B**) combined effect of antibiotics and BSO. Results of ANOVA. Displayed as least squares weighted means and standard errors.

Differences between strains of *E. coli* are also evident from the zones of inhibition tests carried out for a set of 11 antibiotics. Individual *E. coli* were grouped according to their relationship to antibiotics and BSO. Additionally, the effect of co-treatment with antibiotics and BSO on the size of inhibition zone was evaluated. In the first step, the effect of BSO addition on the size of inhibition zones was analyzed. ANOVA revealed no significant differences in the size of inhibition zones after BSO addition; BSO reduced the inhibition zone to 85% of its initial size only (Figure 3A). Accordingly, the combined effect of antibiotics and BSO addition on the inhibition zone was also insignificant; the only significance was caused by a single effect of antibiotics, F(10,198) = 6.5, *p* < 0.001. The highest inhibition zone-sizes were apparent for tetracycline, smallest inhibition zones were apparent for bacitracin (Figure 3B).

**Figure 4:**
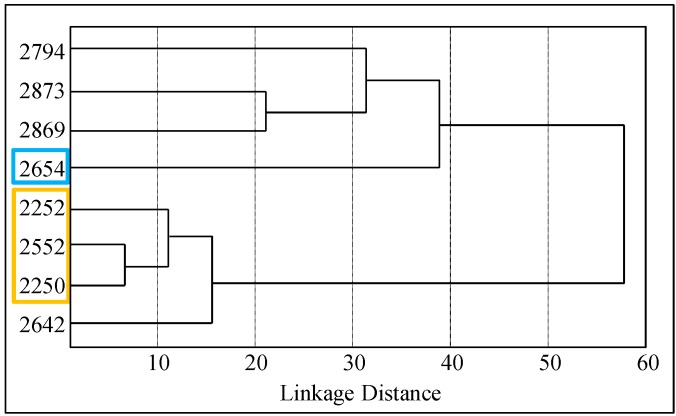
The results of the hierarchical cluster analysis based on the *E. coli* resistance to the antibiotics. Distribution of patients according to the relation to antibiotics. Colors are indicated in patients who had greater resistance (**R**, yellow) or sensitivity (**S**, blue) to antibiotics.

Based on this distribution, we distinguished two diverse groups of isolated *E. coli* strains (Figure 4)—the group, which was based on statistical evaluation of the inhibition zones showed more resistant properties (**R**), and the group, which exhibited higher sensitivity (**S**) towards antibiotics.

Based on distribution we attempted to find the differences in the metabolic pathways of GSH and GSH-related genes. Firstly, we compared the changes in the GSH concentration before and after blocking by using BSO. Then, we focused on changes affecting the kinetics of enzymatic reactions of glutathione synthesis.

The presence and concentration of GSH was determined by three methods: high performance liquid chromatography with electrochemical detection (HPLC-ED), ion-exchange liquid chromatography with UV/Vis detection and MALDI-TOF MS.

### 2.4. Change in the Concentration of GSH

**Figure 5:**
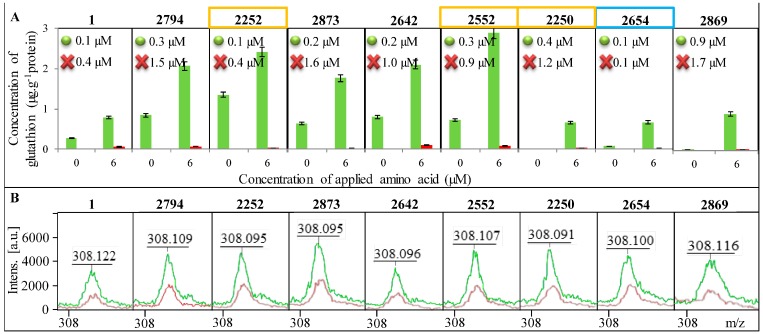
Presence of GSH in individual *E. coli* bacterial strains. Identification of *E. coli* is shown in the upper part of each image. Control variant is always highlighted in green and the variant with the blocked enzyme in red. (**A**) Determination of the concentration of GSH before and after the addition of amino acid precursors. *K*_m_ are inscribed in graphs where the green dot indicates the control *E. coli* a red cross variant of *E. coli* with blocked synthesis of GSH; (**B**) Confirmation of the presence of GSH using MALDI-TOF MS—green curve indicates the control variant and red the blocked one.

In Figure 5A, a change in the concentration of GSH in all tested strains of *E. coli* is shown as well as the increase in concentration after addition of 6 µM amino acids (glycine, glutamic acid and cysteine) serving as substrates for the enzymatic reaction. A significant reduction in GSH concentration after blocking of its synthesis can be seen. The change in GSH concentration after the addition of amino acid precursors was used to calculate *K*_m_, which is shown in each graph of Figure 5A. All tested *E. coli* required a higher concentration of the substrate for the enzymatic reaction after the blocked synthesis of GSH compared to *E. coli* without blocked GSH synthesis. However, the difference in *K*_m_ between individual *E. coli* is considerable. While in *E. coli* from sample 2654 the difference in *K*_m_ between control and the variant with the blocked GCL gene is minimal, in samples 2794, 2873, 2642 and 2250, there was a significant increase in *K*_m_, approximately about 1 µM.

The highest concentration of GSH in *E. coli* was determined in sample No. 2252 and it was 1.4 μg g^−1^ of protein. Concentrations below the limit of detection of the method were determined in samples No. 2250 and No. 2258. In comparison of GSH concentrations in *E. coli* with and without blocked GCL, the variants with added amino acids provided 20–100 times lower concentrations of GSH. In *E. coli* without the addition of amino acids, the concentration of GSH after blocking GCL was more than one thousand times lower. The decreased concentration of GSH after the addition of BSO was confirmed by mass spectra (Figure 5B).

### 2.5. Changes in Gene Expression and Sequence

**Figure 6:**
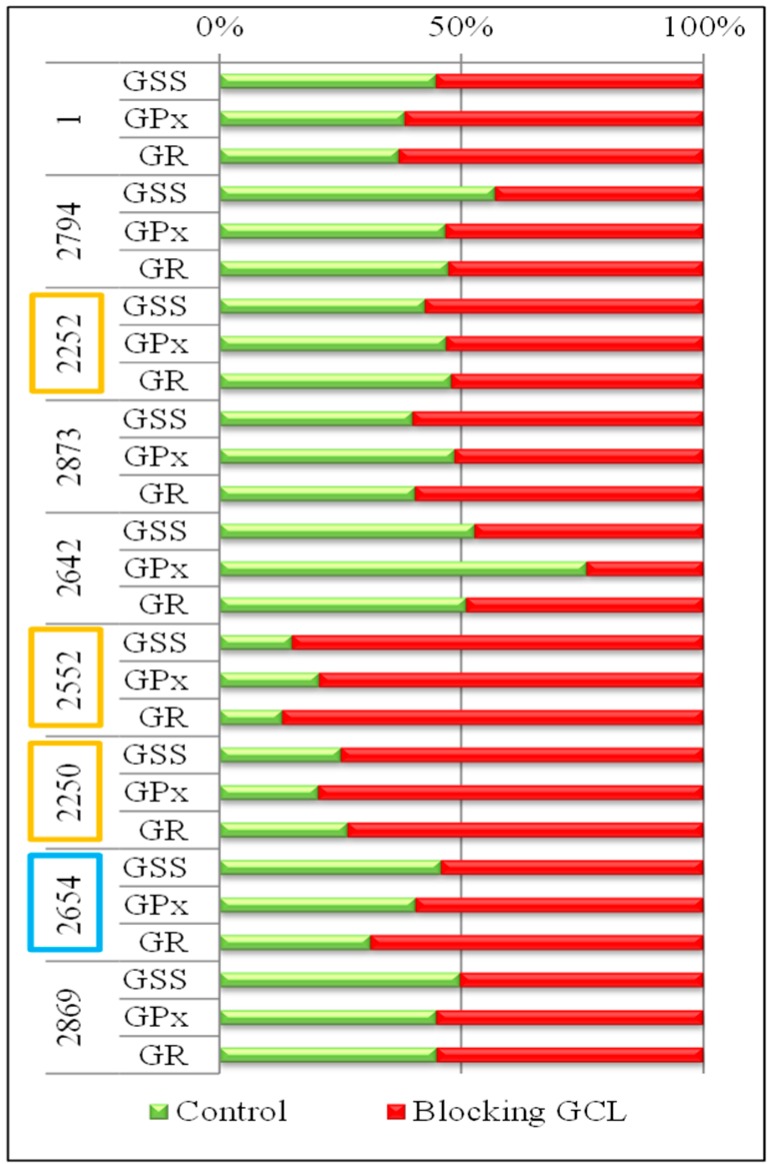
Level of gene expression of *GSS*, *GPx* and *GR* in *E. coli* obtained from the wounds of patients. Comparison of gene expression for the control *E. coli* (green), and *E. coli* with BSO, which blocks the synthesis of GSH on the level of *GCL* gene (red). The overlap of one or the other color over 50% indicates an increased expression compared with the other variant.

The influence of stress factors on cells leads to increased synthesis of GSH and increased expression of enzymes dealing with oxidative stress; (GPx and GR) belongs among these enzymes of the glutathione pathway, and the GCL enzyme, for which activity was blocked with BSO. The GSH synthesis pathway also includes GSS, which is co-responsible for the synthesis of GSH. Differences in expression of these genes (*GSS*, *GPx* and *GR*) in different strains of *E. coli* with blocked GCL and in the control *E. coli* strain (without blocking) pointed to the important biochemical differences between *E. coli* strains isolated from swabs of each patient suffering from bacterial infection (Figure 6). *E. coli* strains from patients marked as No. 2552 and No. 2250, with blocked GCL exhibited at least about 75% higher expression of *GSS*, *GPx* and *GR* genes. In contrast, in patients No. 2794, 2252, 2873 and 2869, a minimum increase in the expression of these genes with the blocked GCL enzyme was identified. It is interesting, that in the majority of *E. coli* samples a comparable influence of blocking on all genes was determined. The difference is evident only in the sample 2642 in the *GPx* gene. For this gene, the expression in the control *E. coli* was approximately 75% higher than in *E. coli* after blocking GCL. These results indicated that the **R** group, represented by samples No. 2552 and No. 2250, showed higher expression of the enzymes of GSH pathway after GCL blockage. However, samples of this group (No. 2252) did not show the same behavior.

Changes in the genetic information of tested *E. coli*, which could have an impact on different parts of the GSH pathway, were determined by sequencing the genes involved in the GSH pathway (*GSS*, *GPx* and *GR*). Mutations were observed for all tested samples from wounds of patients (compared to the laboratory *E. coli*).

On the level of genetic information, significant changes between proposed groups **R** and **S** were found, especially for the *GSS* gene where the changes were the biggest. Samples No. 2252, 2552 and 2250, classified into the **R** group, had only a minimal number of point mutations (in the range of 0–2 point mutations in the gene sequence). In contrast, No. 2654 belonging to the **S** group, showed eight point mutations. In other *E. coli*, the number of *GSS* mutations (6–7) was determined (Table 2). These data pointed at requirements of the *GSS* gene integrity, leading to proper functionality of the enzyme and finally to proper GSH synthesis, which can be partially involved in bacterial resistance, in similar way as in malignant tumors [26].

**Table 2:** Mutations in *GSS* sequences. Position specified in the top line is highlighted bold in the table. Red letters represent the base, which compared with the control *E. coli* possesses a mutation at a given position. The sum of these mutations is given in the last column of the table.

Sample	Position of Mutation						Number of Mutations
	720	753	762	768	780	853	
1	…CG-G- **C**…	…- **T**…	… **C**…	… **T**…	… **A**…	… **A**…	-
2794	…CG-TG**C**…	…-C…	…T…	…C…	…G…	…C…	7
2252	…CG-G- **C**…	…- **T**…	… **C**…	… **T**…	… **A**…	… **A**…	0
2873	…CG-GT**C**…	…-C…	… T…	…C…	…G…	…C…	6
2642	…CG-GT**C**…	…-C…	…T…	…C…	…G…	…C…	6
2552	…CG-GT**C**…	…- **T**…	… **C**…	… **T**…	… **A**…	… **A**…	1
2250	…CGTGT**C**…	…- **T**…	… **C**…	… **T**…	… **A**…	… **A**…	2
2654	…TGTG-**C**…	…GC…	… **T**…	…C…	…G…	…C…	8
2869	…CG-GT**C**…	…-C…	…T…	…C…	…G…	… C…	6

Differences can also be seen between the **R** and **S** group in the *GR* gene on the basis of point mutations in the gene sequence, but were not as significant as in the case of *GSS*. Samples from the **S** group had the highest number of mutations (9) compared to the laboratory *E. coli*, while in the **R** group the number of mutations varied from 3 to 6 (Table 3).

**Table 3:** Mutations in *GR* sequences. Position specified in the top line is in the table highlighted bold. Red letters stand for the base, which compared with the control *E. coli* possesses a mutation at a given position. The sum of these mutations is given in the last column of the table.

Sample	Position of Mutation						Number of Mutations
	678	697	702	712	717	720	
1	… **C**-A-…	… **G**-C…	…-T **A**G…	… **A**…	… **T**…	… **T**…	-
2794	… T -A-…	… **G** TC…	…-T G G…	… G…	… C …	… C …	6
2252	… **C**-A-…	… **G** TC…	…-- **A**G…	… G …	… **T**…	… **T**…	3
2873	… TAG-…	… **G** TC…	…-T G G…	… G …	… C …	… C …	8
2642	… **C**-A-…	… **G** TC…	…-- **A** A…	… G …	… **T**…	… **T**…	4
2552	… T ---…	… **G** TC…	…AT **A** A…	… G…	… **T**…	… **T**…	6
2250	… **C**-A-…	… **G** TC…	…-- **A**G…	… G …	… **T**…	… **T**…	4
2654	… T -TA…	… **G** TC…	…-- G G…	… G …	… C …	… C …	9
2869	… T -AG…	… **G** TC…	…-- GG…	… G …	…C…	… C…	8

For the *GPx* gene, the differences between the **S** and **R** groups based on point mutations in the sequence can also be seen, but not as significant as in case of *GSS*. Samples from the **S** group had three mutations compared to laboratory *E. coli*. The number of mutations in the **R** group is in the range from 1 to 2 (Table 4).

**Table 4:** Mutations in *GPx* sequences. Position specified in the top line is in the table highlighted bold. Red letters stand for the base, which compared with the control *E. coli* possesses a mutation at a given position. The sum of these mutations is given in the last column of the table.

Sample	Position of Mutation		Number of Mutations
	428	485	
1	… **G**C–GG–…	… **A**…	-
2794	… **G**CTGGA…	… T…	3
2252	… **G**CTGG–…	… **A**…	1
2873	… **G**CTGGA…	… T…	3
2642	… **G**CTGG–…	… **A**…	1
2552	… **G**CTGG–…	… T…	2
2250	… **G**CTGG–…	… T…	2
2654	… **G**CTGGA…	… T…	3
2869	… **G**CTGGA…	… T…	3

### 2.6. Statistical Evaluation of Glutathione Parameters

**Figure 7:**
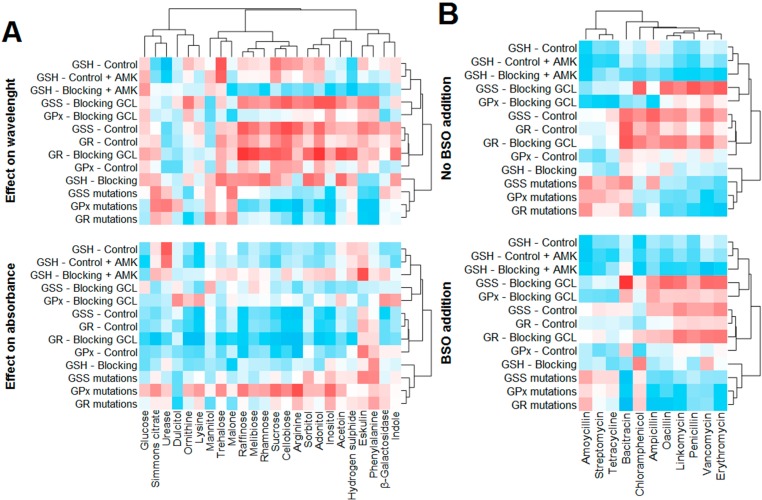
Correlation between oxidative stress-related parameters and biochemical parameters of *E. coli* (**A**) size of inhibition zones for individual antibiotics (**B**). The effect of GSH on biochemical parameters is divided as follows: first, effect on the wavelength maximum of individual parameter and second, the effect on absorbance of individual biochemical parameter. The set of inhibition zones is tested natively and with the addition of BSO.

First, the effect of GSH-related data (including blocking, and mutational analysis) on the tested biochemical parameters of *E. coli* was statistically analyzed. Because the wavelength maxima varied in individual treatments, this parameter was also analyzed in addition to the absorbance of individual biochemical parameters. The overall effect is depicted in Figure 7A. The absorbance of a majority of components decreased with increasing GSH-related parameters (*i.e.*, negative correlation, marked with blue), except for esculin and phenylalanine. Conversely, the increasing number of mutations in *GPx* and *GR* genes caused elevations in the level of the majority of compounds. With regard to the effect on the wavelength, more heterogeneous results were observed. Thus, the GSH-related parameters can be divided as “elevating the wavelength” (GSH, *GPx*, *GR*, and *GSS*), and “reducing the wavelength” (mutations in *GSS*, *GPx* and *GR*). Nevertheless, several compounds expressed different trends; these were increasing together with a number of point mutation in these genes. These include Simmons citrate, urease, mannitol, trehalose, malonate and hydrogen sulfide.

With regard to the effect of GSH-related parameters, antibiotics can be divided into two groups. This is also well evident from the correlation analysis and the cluster analysis (Figure 7B). The so-called “first group” of antibiotics consists of amoxicillin, streptomycin and tetracycline (all those caused the largest inhibition zones, as apparent from Figure 3B). These three antibiotics are characteristic by the fact, that the GSH, GPx, GSS and GR (negative correlation, *i.e.*, smaller size of inhibition zone with increasing level of GSH and GSH-related enzymes) negatively affect the inhibition zones. Also a positive correlation with a number of mutations in *GSS*, *GR*, and *GPx* genes (higher number of mutations causes increase of the inhibition zone size) occurred. The “second” group of antibiotics consists of the remainder, included in the analysis (all those caused rather smaller size inhibition zones, Figure 3B). The inhibition zone of those antibiotics was positively correlated by *GSS*, *GR* and *GPx*, and negatively correlated by the number of the mutations in these genes.

## 3. Experimental Section

### 3.1. Chemicals

BSO, and other chemicals listed in the text were purchased from Sigma-Aldrich (St. Louis, MO, USA) in ACS (American Chemical Society) purity, unless stated otherwise. The deionized water was prepared using reverse osmosis equipment Aqual 25 (Aqual s.r.o., Brno, Czech Republic). The deionized water was further purified by using apparatus Milli-Q Direct QUV equipped with an UV lamp from Millipore (Billerica, MA, USA). The resistance was 18 MΩ. The pH was measured using pH meter WTW inoLab (Weilheim, Germany).

### 3.2. Cultivation of Escherichia coli

*Escherichia coli* (NCTC 13216) was obtained from the Czech Collection of Microorganisms, Faculty of Science, Masaryk University, Brno, Czech Republic. The strain was stored as a spore suspension in 20% (*v*/*v*) glycerol at −20 °C. Prior to use in this study, the strains were thawed and the glycerol was removed by washing with distilled water. The composition of cultivation medium was as follows: tryptone 10 g·L^−1^, yeast extract 5 g·L^−1^, NaCl 5 g·L^−1^, sterilized MilliQ water with 18 MΩ. The pH of the cultivation medium was adjusted at 7.4 before sterilization. The sterilization of the media was carried out at 121 °C for 30 min in sterilizer (Tuttnauer 2450EL, Jerusalem, Israel). The prepared cultivation media were inoculated with bacterial culture in 25 mL Erlenmeyer flasks. After inoculation, the bacterial cultures were cultivated for 24 h on a shaker at 600 rpm and 37 °C.

### 3.3. Preparation of Hospital Samples and Their Cultivation

#### 3.3.1. Wounds Swabs of Patients with Bacterial Infections

Clinical specimens including wound swabs were obtained from Trauma Hospital in Brno, Czech Republic (Table 5). The Ethics Committee of the Trauma Hospital in Brno, Department of Traumatology at the Medical Faculty, Masaryk University of Brno, Czech Republic, approved enrollment of patients into the clinical study. Smears were obtained from a patient with infectious wounds before the medical treatment, during treatment and at regular intervals after completion of the treatment process. Smears were done by rolling motion at the site of skin puncture using a sterile swab sampling. The swab was then placed into a tube with a semi-solid transport medium and carefully sealed and labeled. Marked tubes were immediately handed over to microbiological examination. For analysis, patients aged less than 60 years without diagnosis of coronary artery or peripheral arterial disease, non-smokers, not under corticosteroid treatment or immunosuppressant in regular medication were selected. The swab samples were left in the transport medium intended for the storage of the sample before culturing in the appropriate medium.

#### 3.3.2. Cultivation of Clinical Specimens

The isolation of bacterial strains from hospital samples was performed using selective blood agar. The swab sample was cultivated on blood agar with 10% NaCl, blood agar without other compounds [27], Endo agar [28] and blood agar with amikacin [29]. These Petri dishes was cultivated for 24–48 h at 37 °C. The identification of bacterial strains isolated from hospital samples was done using mass spectrometry MALDI-TOF MS [30].

#### 3.3.3. MALDI-TOF—Confirmation of GSH Presence

Five-hundred µL of *Escherichia coli* cultures, grown overnight, were centrifuged at 14,000× *g* for 2 min, the supernatant was discarded and the pellet was suspended in 300 µL of de-ionized water; then, 900 µL of absolute ethanol was added. After centrifugation at 14,000× *g* for 2 min, the supernatant was discarded and the pellet was air-dried. The pellet was then dissolved in 25 µL of 70% formic acid and 25 µL of acetonitrile and mixed. The samples were centrifuged at 14,000× *g* for 2 min and 1 µL of clear supernatant was spotted in duplicate onto the MALDI target (MTP 384 target polished steel plate; Bruker Daltonics, Bremen, Germany) and air-dried at room temperature.

**Table 5:** Characterization of wounds of patients from whom the swabs were obtained and *E. coli* subsequently cultured.

Sample No.	Origin of Smear of Bacterial Infection	Lesion	DM	Sex	Age	Related Diseases	Duration of Treatment	Antibiotic Therapy
1	laboratory *E. coli* strain	standard	NO	-	-	-	-	-
2794	relapse of perianal fistula	fistula	NO	W	35	severe obesity, * GERD, Dalacine intolerance	28 days	Biseptol
2252	periproctal abscess, ** DM	abscess	YES	M	85	hypertension, ischemic heart disease, myocardial infarction, obesity	4 days	without
2873	decubitus of left heel, insulin therapy in DM	decubitus	YES	M	78	hypertension, prostatic hyperplasia, osteosynthesis	39 days	Augmentin, Amikin, Dalacin
2642	decubitus of sacrum	decubitus	NO	M	83	hyperplasia, incontinence, deep vein thrombosis, immobility	47 days	Biseptol, Ciphin
2552	perianal abscess	abscess	YES	W	43	depression, uro-oncology findings, polyvalent drug allergy	19 days	Augmentin, Ciphin, Metronidazol
2250	periproctal abscess,	abscess	YES	W	43	depression, uro-oncology findings, polyvalent drug allergy	19 days	Augmentin, Ciphin, Metronidazol
2654	varicose ulcer of the right leg	ulcus	NO	W	82	myeloproliferative syndrome, immunosuppression—corticosteroids	1 month	Augmentin
2869	decubitus of left heel, insulin therapy in DM	decubitus	YES	M	78	hypertension, prostatic hyperplasia, osteosynthesis	39 days	Augmentin, Amikin, Dalacin

* GERD—gastroesophageal reflux disease, ** DM—diabetes mellitus.

The mass spectrometry experiments were performed on a MALDI-TOF mass spectrometer Bruker ultrafleXtreme (Bruker Daltonik GmbH, Bremen, Germany) equipped with a laser operating at wavelength of 355 nm with an accelerating voltage of 25 kV, cooled with nitrogen and a maximum energy of 43.2 µJ with repetition rate 2000 Hz in linear and positive mode. The matrix used in the MALDI method was α-cyano-4-hydroxycinnamic acid. The matrix was prepared in TA50 (50% acetonitrile, 0.1% trifluoroacetic acid solution). Working standard solutions were prepared daily by dilution of the stock solutions. One µL of sample was applied on the target and dried under atmospheric pressure and ambient temperature. Then, 1 μL of matrix solution was added on the same spot and dried. A mixture of peptide calibration standards (Bruker, Billerica, Germany) was used to externally calibrate the instrument. The MS spectra were typically acquired by averaging 20 sub spectra from a total of 500 shots of the laser (Smartbeam 2. Version: 1_0_38.5). For identification of bacteria, the linear positive method was used in mass range 1–20 kDa and identification was made by comparing measured mass spectra with mass spectra in database using MALDI Biotyper 3.0. For verification of GSH, the reflector positive mode was used in mass range 0–3000 Da.

### 3.4. Analysis of Biochemical Parameters of E. coli

*E. coli* isolated strains were tested using biochemical detection tests called ENTEROtest 24 (Erba Lachema, Brno, Czech Republic) for the following substances: indole, hydrogen sulfide, lysine, ornithine, urease, arginine, Simmons citrate, malone, phenylalanine, β-galactosidase, inositol, adonitol, cellobiose, sucrose, trehalose, mannitol, acetoin, aesculin, sorbitol, rhamnose, melibiose, raffinose, dulcitol, glucose. Substances were mixed with *E. coli* bacterial cultures (total volume 300 μL). Colorimetric changes were observed after 24 h of incubation in 37 °C in constant shaking.

### 3.5. Growth Curves

The antimicrobial effects of tested compounds were evaluated through the absorbance by using the apparatus Multiskan EX (Thermo Fisher Scientific, Schwerte, Germany). Culture was diluted with LB medium at a wavelength of 620 nm to absorbance 0.1. Cultures were mixed in the microplate with various concentrations of BSO or bacterial cultures alone as a control for measurements. The concentrations of BSO were 0.6125; 1.25; 2.5; 5; 10 and 20 mM. Total volume in the microplate wells was always 300 µL. Measurements were carried out at time 0 and then half-hourly for 24 h at 37 °C and a wavelength of 620 nm.

### 3.6. Inhibition Zones

Agar surface in Petri dishes was covered with a mixture of 100 mL of 24 h grown culture of isolated *E. coli* strains with 3 mL of LB medium. From the fabrics, which were made by VUP Medical, PLC in Brno, were cut out circles with diameter of 1 cm and were dipped in a solution of 1 mM antibiotics (erythromycin, penicillin, lincomycin, tetracycline, amoxicillin, ampicillin, streptomycin, bacitracin, vancomycin, chloramphenicol, oxacillin). Dishes were incubated in a thermostat present at 37 °C for 24 h.

### 3.7. Determination of the Total Glutathione and Calculation of Enzyme Activity

The rate of the enzymatic reaction was expressed by the Michaelis constant (*K*_m_), which was calculated using the Michaelis-Menten equation.

#### 3.7.1. Preparation of a Lysate from *Escherichia coli*

Five mL of bacterial culture of *E. coli* was collected before and after six hours of cultivation with BSO. Bacterial culture was divided equally into three 2 mL microtubes, which were centrifuged (Centrifuge 5417R, Eppendorf, Hamburg, Germany) 15 min at 3900× *g* and 25 °C. After removing the supernatant, 2 mL of washing buffer A (80 mmol·L^−1^ KCl, 70 mmol·L^−1^ NaCl, 0.15 mmol·L^−1^ MgCl_2_, 10 mmol·L^−1^ HEPES-Na and 0.1 mmol·L^−1^ EDTA, pH 7.55) were added to microtubes with bacterial cells and the cells were resuspended and then centrifuged under the same conditions as in the previous case.

After removing the supernatant 2 mL of buffer B (80 mmol·L^−1^ KCl, 70 mmol·L^−1^ NaCl, 0.15 mmol·L^−1^ MgCl_2_, 10 mmol·L^−1^ HEPES-Na, 10 mmol·L^−1^ glucose, pH 7.55 s CNDB 0.1 mg·L^−1^) were added to the cells. Samples of bacterial cells were placed in a Thermomixer Comfort (Eppendorf, Germany), where they were incubated for 40 min at 300 rpm and 37 °C. After incubation, centrifugation was carried out in the same way as in the previous cases. After removing the supernatant, the samples were concentrated to one 2 mL microtube, which was supplemented with buffer B (2 mL).

Two mL of bacterial culture was thoroughly resuspended and divided into 14 equal aliquots (100 µL each) into microtubes, which were centrifuged. After centrifugation, the supernatant was removed. The pellet of the bacterial culture was subsequently immersed for 1 min into liquid nitrogen and then 500 µL of the amino acid substrate (glycin, glutamic acid and cystein in buffer B) at a concentration of 0; 0.25; 0.5; 0.75; 1; 1.25; 1.5; 1.75; 2; 2.25; 2.5; 2.75; 3 and 6 µM was added to the each sample. After thorough mixing, the samples were placed for 2 min in an ultrasonic bath Sonorex digital 10P (Bandelin, Berlin, Germany), then were shaken for 30 min at 1400 rpm and 25 °C. Finally, the samples were centrifuged for 2 min at 25 °C and 25,000× *g*. The resulting supernatants were immediately analyzed.

#### 3.7.2. Determination of GSH Concentration by High-Performance Liquid Chromatography with Electrochemical Detection

HPLC-ED system consisted of two solvent delivery pumps operating in the range of 0.001–9.999 mL/min (Model 582 ESA Inc., Chelmsford, MA, USA), Zorbax eclipse AAA C18 (150 × 4.6; 3.5 μm particles, Agilent Technologies, Santa Clara, CA, USA) and a CoulArray electrochemical detector (Model 5600A, ESA, Chelmsford, MA, USA). The electrochemical detector includes one flow cell (Model 6210, ESA, USA). Each cell consists of four working carbon porous electrodes, each one with auxiliary and dry Pd/H2 reference electrodes. Both the detector and the reaction coil/column were thermostated. The sample (20 μL) was injected using an autosampler (Model 542 HPLC, ESA, USA). Samples were kept in the carousel at 8 °C during the analysis. The column was thermostated at 32 °C. The mobile phase consisted of 80 mM TFA (A) and methanol (B). The compounds of interest were separated by the following linear gradient: 0 → 1 min (3% B), 1 → 2 min (10% B), 2 → 5 min (30% B), 5 → 6 min (98% B). Mobile phase flow rate was 1 mL·min^−1^, working electrode potential was 900 mV. Time of analysis was 45 min.

#### 3.7.3. Determination of GSH Concentration by Ion-Exchange Liquid Chromatography

For identification of GSH the ion-exchange liquid chromatography with post column derivatization by ninhydrin and the absorbance detector operating in the VIS range at 570 nm was employed. Glass column tempered to 60 °C with inner diameter of 3.7 and 350 mm length was filled manually with strong cation exchanger in sodium cycle LG ANB with approximately 12 μm particles and 8% porosity. The elution mobile phase (pH 2.7) contained 11.11 g of citric acid, 4.04 g of sodium citrate, 9.25 g of NaCl, 0.1 g of sodium azide and 2.5 mL of thiodiglycol per liter of solution, using the flow rate of 0.25 mL·min^−1^. Other experimental conditions were used as previously published [31].

### 3.8. RNA Isolation, RT-PCR and Gel Electrophoresis

Bacterial cultures were centrifuged at 6000× *g* at 20 °C for 10 min and the pellets were resuspended in 100 µL of PBS buffer, 100 µL of Tissue Lysis Buffer and 0.1 µL of RNase inhibitors (Life Technologies, Pitam Pura, India). This volume was pipetted into the sample tube from MagNA Pure Compact RNA Isolation Kit (Roche, Basel, Switzerland), and inserted with other instruments on the appropriate place in the machine. In the second row of the machine, the vials with 20 µL of DNAse were inserted. Next steps were carried out according to the manufacturer’s instructions (“RNA Cell” protocol MagNA). Obtained RNA concentration was measured using Infinite M 200 pro (Tecan, Grödig, Austria) and diluted to 10 μg mL**^−^**^1^. The RNA was then converted to cDNA using High-Capacity cDNA Reverse Transcription Kit (Life Technologies, India), using random hexamers. The reaction profile was as follows: 25 °C for 10 min, 37 °C for 120 min and 85 °C for 5 min.

Genes were amplified using polymerase chain reaction (PCR). The final volume of the PCR reaction mixture was 25 μL containing 14.42 μL of sterile water, 2.5 μL of 1× Taq reaction buffer, 0.5 μL of 100 mM dNTP, 1.25 μL of forward primer, 1.25 μL of reverse primer, 0.085 μL of Taq DNA polymerase and 5 μL of cDNA. The reaction profile was as follows: 30 cycles of 94 °C for 3 min, 54 °C for 30 s and 72 °C for 30 s and a final extension at 72 °C for 4 min. The amplification was carried out using Mastercycler ep realplex4S (Eppendorf AG, Hamburg, Germany) and 219, 151, and 189 bp fragments were obtained.

The 16S gene was amplified using PCR. The sequences of forward and reverse primers were 5'-GAGTTTGATCCTGGCTCAG-3' and 5'-GGTTACCTTGTTACGACTT-3' respectively. The final volume of the PCR reaction mixture was 25 μL containing 14.42 μL of sterile water, 2.5 μL of 1× Taq reaction buffer, 0.5 μL of 100 mM dNTP, 1.25 μL of forward primer, 1.25 μL of reverse primer, 0.085 μL of Taq DNA polymerase and 5 μL of cDNA. The reaction profile was as follows: initial denaturation at 94 °C for 4 min, 30 cycles of 94 °C for 30 s, 52 °C for 30 s and 72 °C for 1.5 min and a final extension at 72 °C for 10 min. Finally, a 1500 bp fragment was obtained.

DNA was mixed with loading buffer and then pipeted into the wells and 2% agarose gel electrophoresis run in 1× TAE buffer with ethidium bromide for 160 min, 60 V. The bands were visualized by UV transilluminator at 312 nm (VilberLourmat, Marne-la-Valle’e Cedex, France) and band intensities were quantified and analyzed by Carestream Molecular Imaging Software and *in vivo* Xtreme Imaging System (Carestream, Rochester, NY, USA) and normalized to 16S control.

### 3.9. DNA Sequencing

For sequencing reaction, the DTCS Quick Start Kit (Beckman Coulter, Brea, CA, USA) was used. To 20 µL sequencing reaction mixture, 13 ng of DNA fragment for *gpo* and *gr* genes or 16 ng for *gshA* gene, 0.75 µL of 10 µM forward primer, 8 µL of DTCS Quick Start Master Mix and H_2_O (sterile, ACS purity, Sigma-Aldrich, USA) were added and the mixture was transferred to the cycler (Eppendorf, Hamburg, Germany). The conditions of 30 cycle-reaction were as follows: 96 °C for 20 s; 50 °C for 20 s and 60 °C for 4 min. For purification of sequencing product CleanSEQ kit (Beckman Coulter, USA) was used. 20 µL of sequencing product was transferred in microcentrifuge tube and 10 µL of CleanSEQ magnetic particles (MPs) and 62 µL of 85% ethanol (*v*/*v*, with water) were added to the product. The suspension was mixed with the pipette and the fluorescently marked DNA bound to MPs surface. The microcentrifuge tube with the mixture was placed on a magnetic stand (Dynal, Oslo, Norway) for 5 min. The solution was pipetted out and MPs was twice washed with 100 µL of 85% ethanol (*v*/*v*, with water). After removing ethanol, the microcentrifuge tube was transferred from the magnetic stand and 40 µL of Sample Loading Solution was added to MPs. After mixing, MPs were placed on the magnetic stand for 5 min. Then purified samples were transferred to the plate in Sample Loading Solution and DNA sequencing was performed using Genetic Analysis System CEQ 8000 (Beckman Coulter, USA). After denaturation at 90 °C for 2 min, fluorescently marked DNA fragments were separated in a 33 cm long capillary with 75 μm internal diameter (Beckman Coulter, USA), which was filled with a linear polyacrylamide denaturing gel (Beckman Coulter, USA). The separation was run at capillary temperature of 50 °C and voltage of 4.0 kV for 85 min.

### 3.10. Statistical Analysis

Data were processed using MICROSOFT EXCEL (Redmond, WA, USA). Results are expressed as mean ± standard deviation (SD) unless noted otherwise. The experiments were carried out in triplicates. Half-maximal concentrations (IC_50_) were calculated from logarithmic regression of sigmoidal dose-response curve. To reveal differences between categorical predictors, multivariate ANOVA was used, followed by Tukey’s *post hoc* test. To analyze dependences between continuous variables, Pearson correlation was used followed by hierarchical clustering with Ward’s method. Unless noted otherwise, *p* < 0.05 was considered significant. Software STATISTICA (data analysis software system), version 10.0 (Tulsa, OK, USA) was used for data processing. 

## 4. Conclusions

In this work, the differences in *E. coli* derived from swabs from infectious wounds of patients were evaluated, depending on the blocking GCL using BSO. For all the tested *E. coli* strains 24IC_50_ for BSO in the range 15 to 25 mM was determined. No differences in qualitative protein composition before and after the blocking of the synthesis of GSH using mass spectrometry analysis were observed. Significant differences were observed in the resistance to commonly used antibiotics, and based on this resistance, *E. coli* samples can be grouped as more sensitive and more resistant. Differences between these two groups such as the kinetics of synthesis of glutathione, gene expression and gene sequence *GSS*, *GR* and *GPx* were observed. “More sensitive” *E. coli* strains exhibited the same kinetics of GSH synthesis in blocking of GCL (*K*_m_ 0.1 µM) as without blocking, in contrast to the more resistant *E. coli* samples, which showed differences in *K*_m_ between 0.3 and 0.8 µM. With regards to the changes in gene expression, “more resistant” *E. coli* exhibited roughly 75% higher expression of all tested genes in case of BSO application. Differences in the rate of mutations were observed in sequences of these genes, where more resistant *E. coli* exhibited fewer mutations in *GSS*, *GR*, and *GPx*. In summary, these glutathione-derived parameters reflect the severity of resistance of various *E. coli* samples with potential diagnostic applications, which can then lead to elevation of treatment efficiency.
